# Peripheral and Central Nervous System Immune Response Crosstalk in Amyotrophic Lateral Sclerosis

**DOI:** 10.3389/fnins.2020.00575

**Published:** 2020-06-16

**Authors:** Zhouyang Liu, Xi Cheng, Shanshan Zhong, Xiuchun Zhang, Chang Liu, Fangxi Liu, Chuansheng Zhao

**Affiliations:** ^1^Department of Neurology, The First Hospital of China Medical University, Shenyang, China; ^2^Stroke Center, The First Hospital of China Medical University, Shenyang, China

**Keywords:** amyotrophic lateral sclerosis, central nervous system immune response, crosstalk, neuroinflammation, peripheral immune response

## Abstract

Amyotrophic lateral sclerosis (ALS) is a fatal neurodegenerative disease characterized by muscle weakness due to the degeneration of the upper and lower motor neurons. Neuroinflammation is known as a prominent pathological feature of ALS. Although neuroinflammation cannot trigger ALS, activated central nervous system (CNS) microglia and astrocytes, proinflammatory periphery monocytes/macrophages and T lymphocytes, and infiltrated monocytes/macrophages and T lymphocytes, as well as the immunoreactive molecules they release, are closely related to disease progression. The crosstalk between the peripheral and CNS immune components mentioned above significantly correlates with survival in patients with ALS. This review provides an update on the role of this crosstalk between the CNS and peripheral immune responses in ALS. Additionally, we discuss changes in the composition of gut microbiota because these can directly or indirectly influence this crosstalk. These recent advances may well provide innovative ways for targeting the molecules associated with this crosstalk and breaking the current treatment impasse in ALS.

## Introduction

Amyotrophic lateral sclerosis (ALS) is a fatal neurodegenerative disease featuring the progressive degeneration of upper motor neurons (MNs) in the motor cortex and lower MNs in the brainstem and spinal cord, which leads to muscle weakness in the earliest stage of the disease, followed by a gradual loss of all muscle control and, eventually, death (Brown and Al-Chalabi, 2017). The vast majority of ALS cases are sporadic (sALS); approximately only 10% of cases are familial ALS (fALS) that are caused by genetic mutations (Renton et al., 2014; Rowland and Shneider, 2001). Gene mutations in Cu/Zn superoxide dismutase (SOD1) (Rosen et al., 1993; Rosen, 1993), TAR DNA binding protein (TARDBP) (Sreedharan et al., 2008), and chromosome 9 open reading frame 72 (C9orf72) (Renton et al., 2011) are responsible for most cases of fALS. Of these, SOD1 was the earliest discovered genetic cause of ALS. Soon, researchers found that the expression of high levels of human SOD in transgenic mice could cause MN diseases similar to human ALS (Gurney et al., 1994). As the clinical manifestations and pathological processes of fALS and sALS are similar (Andersen et al., 1997; Li et al., 1988), the data derived from studies of the SOD1 mouse model are considered to be of value in unraveling the pathologies of both fALS and sALS (Phatnani et al., 2013). Amyotrophic lateral sclerosis is of particular concern because, up to now, only two approved drugs, riluzole and edaravone, have been shown to be clinically effective. Riluzole only prolongs median survival by 2–3 months (Bucchia et al., 2015; Miller et al., 2012). Edaravone only slightly improves patients’ motor function scores (Kosuge et al., 2020; Okada et al., 2018; Sawada, 2017).

The failure of various therapeutic tools can be largely attributed to an incomplete understanding of the pathological basis of ALS. As it is such a prominent feature of many neurodegenerative diseases, including ALS, neuroinflammation has attracted more and more attention. Neuroinflammation is characterized by activated central nervous system (CNS) microglia and astroglia, proinflammatory peripheral lymphocytes, and macrophages and has been observed in both patients and animal disease models (Beers and Appel, 2019; Beers et al., 2006; Beers et al., 2008; Chiu et al., 2009; Lincecum et al., 2010; McGeer and McGeer, 2002; Zhao et al., 2017). Amyotrophic lateral sclerosis is considered as a heterogeneous disease because of the variable progression rates and survival time (Appel et al., 2010; Beers et al., 2017; Zhao et al., 2013). Emerging evidence has revealed that neuroinflammation participates toward this heterogeneity (Beers and Appel, 2019; Frakes et al., 2014; Rusconi et al., 2017).

The CNS was traditionally believed to be immunologically privileged because peripheral immune components were not allowed to infiltrate into the CNS (Appel et al., 2010; Carson et al., 2006). However, there is now convincing evidence indicating that immune responses can and do occur in CNS and that these actively communicate with immune responses occurring in the periphery (Appel et al., 2010; Bechmann, 2005; Carson et al., 2006; Ransohoff et al., 2003; Wekerle, 2006). Here, we have adopted an innovative approach by classifying the immune components from the perspective of both CNS immunity and peripheral immunity in ALS rather than dividing them according to their innate and adaptive immunity origins. This spatial classification method makes the visualization of this complex immune network easier to understand. First, we will review the characteristics of CNS immunity in ALS. Second, we will describe the impairment of the blood–CNS barrier (B-CNS-B) in ALS rodent models and ALS patients, which may lead to an increased interaction between CNS immunity and peripheral immunity, which may affect neuroinflammatory responses and thus on the severity of neurodegeneration (Mosley et al., 2012; Stolp and Dziegielewska, 2009). Third, we will examine the role of peripheral immunity in ALS and its complicated crosstalk with CNS immunity. Finally, we will include the gut microbiota into the category of immune-related component, in terms of ALS neuroinflammation and discuss its interaction with the multiple factors participating in the crosstalk.

Previous investigations of neuroinflammation in ALS have mainly focused on the disease’s CNS pathology. Nevertheless, much less is known about the role of the peripheral immunity and its interaction with CNS immunity in the progression of ALS. This review aims to fill this gap and reexamine ALS neuroinflammation from a more holistic perspective, which will help us better understand its pathological basis and thus find the most valuable therapeutic targets in the entire neuroinflammation system.

## Microglia

**FIGURE 1:**
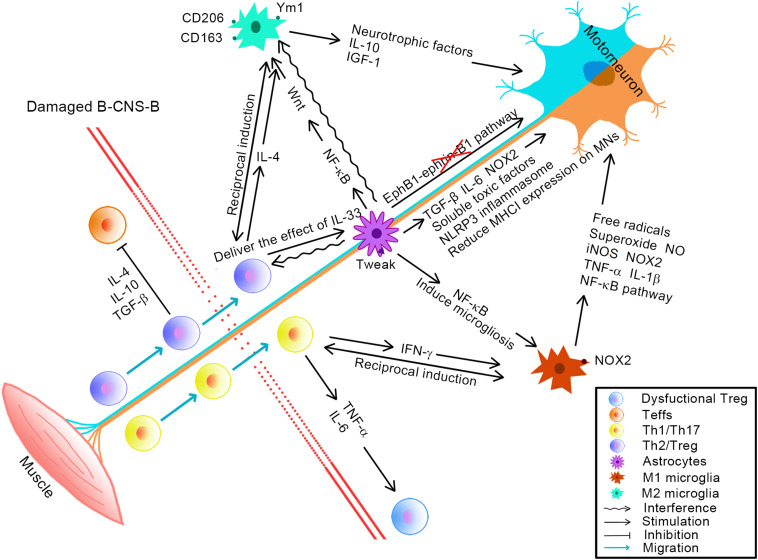
Schematic diagram of the major pathophysiological events in ALS, in terms of microglia, astrocytes, and CD4^+^ T cells. The upper left portion of the diagram depicts the neuroprotective effects of neuroinflammation in ALS. Anti-inflammatory T cells (T_H_2/Treg) migrate from the periphery to the CNS, where they interact with microglia (M2 subtype) and astrocytes to protect motor neurons. The lower right portion of the diagram depicts the neurotoxic effects of neuroinflammation in ALS. Proinflammatory T cells (T_H_1/T_H_17) migrate from the periphery to the CNS, where they interact with microglia (M1 subtype) and astrocytes to damage motor neurons.

Microglia, as resident immune cells of CNS, make great contributions to CNS development, homeostasis, injury, and repair. Microglia are able to interact with a variety of CNS immune components as well as peripheral immune components that have infiltrated into the CNS, making themselves the focus of multiple crosstalk events (Figure 1).

These cells constantly monitor their surroundings and interact closely with other nearby cells. In ALS autopsy specimens, it has been found that reactive microglia, coupled with accumulating T cells, are abundant in the anterior horn of the spinal cord, the corticospinal tract, the motor nuclei of the brain stem, and the motor cortex (Kawamata et al., 1992). Later, another study, based on postmortem tissue, showed that T cells are present in spinal cord specimens from ALS patients (Engelhardt et al., 1993). According to these two results, we speculate that there may well be some dialogue between microglia and infiltrated T cells. Subsequently, convincing data from living ALS sufferers have supported the above observations. Images of ^18^F-DPA-714 positron emission tomography (PET) and [^11^C](R)-PK11195 PET of the brain of ALS patients both reveal the presence of extensive microglia activation (Corcia et al., 2012; Turner et al., 2004). The imaging principle is that ^18^F-DPA-714 and PK11195 are ligands of the 18-kDa translocator protein and the so-called “peripheral benzodiazepine binding site” expressed by activated microglia, respectively. Studies conducted in ALS animal models have also shown that activated microglia can be observed prior to disease onset, and their numbers increase as the disease progresses (Alexianu et al., 2001; Henkel et al., 2006; Zhao et al., 2013). Microglia can exist in a variety of phenotypic states across a continuum, with two extremes, an M2 protective phenotype that produces anti-inflammatory cytokines as well as neurotrophic factors, and an M1 toxic phenotype, which is able to produce reactive oxygen species (ROS) and proinflammatory cytokines (Benner et al., 2008; Gendelman and Appel, 2011). Therefore, whether microglia play a proinflammatory or anti-inflammatory role is influenced by the surrounding cells, because these affect the phagocytic and antigen-presenting properties of these versatile cells (Philips and Robberecht, 2011).

Great strides have been made in clarifying the microglial contribution to ALS; these rapidly followed in the wake of recent discoveries about the spectrum between the two extreme activation states of microglia (M1 and M2). In the presymptomatic disease, microglial cells have been shown to overexpress the anti-inflammatory cytokine, interleukin 10 (IL-10); importantly, it was reported that blocking IL-10 increased inflammation and accelerated clinical onset, whereas overexpressing IL-10 in microglia by gene therapy means delayed disease onset and improved survival of ALS mice; thus, targeted overexpression of IL-10 in microglia may have therapeutic potential in human ALS (Gravel et al., 2016; Figure 1).

In the early stage of the slow progression of the disease, M2 subtype markers, CD163, CD206, and Ym1, were observed to be up-regulated in the spinal cord of ALS mice, suggesting that at least some microglia were displaying an M2 phenotype at this time (Figure 1). As the disease turns toward its more rapid progression stage, mSOD1 microglia (microglia overexpressing mutant superoxide dismutase) in ALS mice spinal cord showed the M1 phenotype, accompanied by increased levels of NOX2 (a marker of M1) (Beers et al., 2011a, b; Liao et al., 2012; Figure 1). When microglia isolated from different stages of the disease were cocultured with MNs, *ex vivo*, the early M2 microglia exerted neuroprotective effects and increased the survival rate of MNs, whereas microglia, isolated from the late disease stage, which were more M1 subtype–like, were toxic to MNs (Liao et al., 2012). These data provide a more comprehensive understanding and analysis of the temporal transformation of the activation states of microglia during the pathological progression of ALS.

The toxic effects of M1 microglia on MNs have been confirmed in many studies. Following lipopolysaccharide (LPS) activation (LPS is an effective M1 stimulant), microglia are capable of releasing free radicals, which may initiate MN damage by increasing the susceptibility of the MN AMPA/kainate receptor to the excitotoxic effects of glutamate (Zhao et al., 2004; Figure 1). Furthermore, LPS-activated mSOD1^G93A^ microglia (i.e., microglia derived from mice overexpressing mutant human SOD1^G93A^) released more superoxide, more nitric oxide (NO), and less insulinlike growth factor 1 (IGF-1), which induced more MN death and reduced the number and length of neurites in microglia/MN cocultures, as compared with LPS-treated nontransgenic microglia (Xiao et al., 2007; Figure 1).

More and more evidence has confirmed the specific role of mutated proteins in driving this increased toxicity driven by M1 type microglia. A recent *in vivo* study has demonstrated that the expression of mSOD1^G93A^ led to activated and neurotoxic microglia and induced more neuronal death (Beers et al., 2006). Interestingly, when the G85R mutation of SOD1 (a disruption that leads a protein without any enzymatic activity) was knocked down in microglia/macrophages, a prolonged early and late disease phase was observed, whereas suppression of G37R (a mutant SOD1 with full dismutase enzymatic activity) expression in the same cells affected only the late phase (Wang et al., 2009). Besides, mutant forms of TDP-43 are involved in activating microglia and up-regulating the expression of proinflammatory factors, such as NOX2, tumor necrosis factor α (TNF-α), and IL-1β (Zhao W. et al., 2015; Figure 1). Recently, RNA-seq analysis identified that inflammatory processes were significantly elevated in ALS, with TNF being found to be a major pathway regulator of these processes (Brohawn et al., 2016). Mutated proteins, SOD1 and TDP-43, induced the selective activation of nuclear factor κB (NF-κB), a master regulator of inflammation. Subsequently, researchers found that the up-regulation of NF-κB signaling in wild-type microglia induced gliosis and MN death, both *in vitro* and *in vivo*, and the down-regulation of NF-κB signaling in microglia rescued MNs from microglial-mediated death *in vitro* and prolonged survival in ALS mice by weakening proinflammatory microglial activation. These experiments therefore indicate that microglia-induced MN death in ALS is executed via the classical NF-κB pathway (Frakes et al., 2014; Zhao W. et al., 2015; Figure 1).

A recent study has shown that the level of IGF-1 was higher in microglia isolated from the spinal cord of SOD1^G93A^ mice, as compared with wild-type microglia (Chiu et al., 2008; Figure 1). The protective effect of IGF-1 on MNs has been proven in animal models of ALS. Delivery of IGF-1, via viral vectors, has been shown to increase MN survival, improve motor function and prolong the life span of ALS mice (Dodge et al., 2008; Lepore et al., 2007). In that study, it was noted that IL-4 promoted the survival of MNs, by inducing a switch from the M1 to the M2 phenotype, characterized by a reduction in the release of free radicals and NO, while increasing the secretion of IGF-1 in microglia/MNs cocultures (Zhao et al., 2006). Interleukin 4 is able to skew microglia toward expressing a group of genes normally encoded by embryonic microglia, and this phenotypic acquisition results in a general amelioration of clinical outcomes during the early slowly progressive stage of ALS (Rossi et al., 2018). Additionally, the M2 phenotype is also capable of inducing regulator T cells (Tregs); these cells are characterized by their strong suppressive activities (Savage et al., 2008; Figure 1).

## Astrocytes

As the most abundant glial cells in CNS, astrocytes are endowed with a variety of abilities that make them key players in the progression of ALS. These include the ability to regulate the immune response in CNS, the possibility to induce and maintain blood–brain barrier (BBB), and the capability to secrete cytokines, chemokines, and neurotrophic factors (Dong and Benveniste, 2001; Farina et al., 2007). In addition to the activation of microglia, astrocyte activation is also a typical hallmark of ALS (Bowerman et al., 2015). In SOD1 animal models, astrogliosis was detected and the increase in the number of activated astrocytes occurred in parallel to the loss of MNs (Hall et al., 1998; Levine et al., 1999). In ALS cases, activated astrocytes have been found throughout the cerebral gray matter and the spinal cord (Nagy et al., 1994; Schiffer et al., 1996). Following activation, astrocytes were critically involved in neuroinflammation-mediated neurotoxicity in a variety of ways (Johann et al., 2015; Philips and Robberecht, 2011). In addition to interacting directly with MNs, activated astrocytes can also communicate with neighboring microglia within the CNS. In addition, astrocytes can regulate BBB permeability and communicate with the infiltrating peripheral immune components (Farina et al., 2007). These behaviors of astrocytes may directly or indirectly affect disease progression.

## Direct Effects of Astroglia

Astrocytes themselves can directly influence the fate of MNs. *In vitro*, rodent astrocytes with mutated SOD1 selectively killed spinal primary and embryonic mouse stem cell–derived MNs by releasing soluble toxic factors, while leaving undamaged spinal GABAergic or dorsal root ganglion neurons or embryonic stem cell–derived interneurons (Nagai et al., 2007; Figure 1). That same year, another study using a murine embryonic stem cell-based ALS model reported similar results. These latter investigators found that, whether or not MNs carried the mutated SOD1^G93A^ gene, they exhibited neurodegenerative characteristics when cocultured with SOD1^G93A^ astrocytes (Di Giorgio et al., 2007).

*In vivo* experiments on animal models subsequently confirmed the above conclusion. Conditioned media derived from primary mouse astrocytes expressing SOD1^G93A^ was slowly introduced into the spinal cord of healthy rats. After this treatment, these healthy rats developed motor dysfunction and astrogliosis in their spinal cords, evidence that soluble toxic factors known to be released *in vitro* from ALS astrocytes could evoke ALS-like symptoms *in vivo* (Ramírez-Jarquín et al., 2017; Figure 1). However, diminishing the expression of the SOD1^G93A^ mutation in astrocytes could effectively alleviate microglial activation and slow disease progression (Yamanaka et al., 2008). In ALS, there is an intricate interplay between astrocytes and MNs that profoundly affects each cellular population. The transforming growth factor β (TGF-β) signaling pathway was identified as one of the signaling pathways that may lead to MN degeneration (Phatnani et al., 2013; Figure 1). Astrocytes derived from either ALS mice or ALS patients displayed a reduced expression of major histocompatibility complex class I (MHCI) molecules on MNs, which makes MNs more sensitive to astrocyte-mediated neurotoxicity, leading to more MN deaths. After increasing the expression of MHCI on MNs, MNs were able to evade astrocyte-mediated neurotoxicity, and both the clinical performance and the survival rate of ALS mice improved. The fundamental mechanism underlying these effects may be the binding of HLA-F (a single MHCI molecule) to its ligand (the killer cell immunoglobulin-like receptor KIR3DL2) (Song et al., 2016; Figure 1). A recent study found that the TNF-like weak inducer of apoptosis (Tweak) was aberrantly expressed in astrocytes in the spinal cord of SOD1^G93A^ mice, which could stimulate astrocyte proliferation, astrocytic IL-6 secretion and induce MN death *in vitro* (Bowerman et al., 2015; Figure 1). Ephrin type B receptor 1 (EphB1) can activate an anti-inflammatory and neuroprotective response in astrocytes; however, the EphB1-ephrin-B1 pathway did not function properly in SOD1^G93A^ mice or in astrocytes derived from stem cells in ALS patients (Tyzack et al., 2017; Figure 1). NLRP3 inflammasome and IL-1β were detected in the spinal cord of SOD1 mice at a presymptomatic stage, and their levels increased as the disease progressed. Furthermore, a high expression of the NLRP3 inflammasome, IL-18, and active caspase 1 were also detected in postmortem tissues of ALS patients. Importantly, the NLRP3 inflammasome and related components are thought to be derived primarily from astrocytes in the spinal cord, indicating that the astrocyte-derived NLRP3 inflammasome contributes to neuroinflammation in ALS (Johann et al., 2015; Figure 1).

Researchers have conducted similar studies on human specimens. Spinal MNs derived from human embryonic stem cells were susceptible to the toxic effects mediated by SOD1 mutated astrocytes (Di Giorgio et al., 2008). Motor neurons were sensitive to the toxic effects mediated by human induced pluripotent stem cell (iPSC)-derived astrocytes from ALS patients with mutated C9orf72, which may be triggered by soluble factors (Birger et al., 2019; Figure 1). When the human primary astrocytes expressing mutated SOD1 were cocultured with the hESC-derived MNs, the astrocytes activated NOX2 and increased the inflammatory response, and this enhanced the toxic effect on MNs (Marchetto et al., 2008; Figure 1). Compared with healthy controls, levels of IL-6 in astrocyte-derived exosomes from sALS patients were elevated and positively correlated with the rate of disease progression (Chen et al., 2019; Figure 1).

## Indirect Effects of Astroglia

Activated astrocytes are able to undergo a crosstalk with the CNS immune components and the infiltrated peripheral immune components, such as microglia and T cells. Local enrichment of normal astrocytes in the cervical spinal cord of SOD1^G93A^ rats decreased microglial proliferation, reduced the disease burden, and extended the animals’ survival (Lepore et al., 2008). Moreover, local enrichment of SOD1^G93A^ astrocytes in the cervical spinal cord of WT rats induced host astrocytosis and microgliosis, subsequently leading to MN death and motor dysfunction in the host (Papadeas et al., 2011; Figure 1). Astrocytes have been demonstrated to amplify the neuroprotective and neurotoxic effects of microglia in the presymptomatic phase and the symptomatic phase, respectively. In the presymptomatic phase, astrocytic NF-κB activation in SOD1 models induced a Wnt- dependent microglial activation, delaying motor symptoms. However, in the symptomatic phase, NF-κB activation in astrocytes promoted microglia proliferation and thus accelerated disease progression. The transition of the astrocytic effect corresponds to the shift in the microglial phenotype, suggesting that astrocytes are important regulators of microglia activation and neuroinflammation in ALS (Ouali Alami et al., 2018; Figure 1). A recent study showed that TGF-β1 expression was up-regulated in astrocytes of murine and human ALS. This astrocyte-specific overproduction of TGF-β1 was thought to accelerate disease progression by interfering with the neuroprotective effects of microglia and T cells, in SOD1^G93A^ mice (Endo et al., 2015; Figure 1). Injection of IL-33 into the abdominal cavity of female SOD1 mice was able to (1) decrease the proportion of CD4^+^ and CD8^+^ T cells in their spleen and lymph nodes, (2) reduce the activation of astrocytes in their spinal cord, and (3) delay the onset of disease. *In vitro* experiments have shown that this beneficial effect of IL-33 is most likely not mediated by any direct action on MNs and astrocytes but by the delivery of its effects via peripheral T cells (Korhonen et al., 2019; Figure 1).

## Blood–Central Nervous System Barrier

The B-CNS-B consists of separate components, that is, the BBB, the blood–spinal cord barrier (BSCB), and the blood–cerebrospinal fluid barrier (Ballabh et al., 2004; Bradbury, 1985; Dermietzel and Krause, 1991; Garbuzova-Davis and Sanberg, 2014; Pardridge, 1991; Pardridge, 1999; Vorbrodt and Dobrogowska, 2003), which limit the entry of toxic circulating molecules, blood cells, and pathogens into the CNS (Montagne et al., 2017; Sweeney et al., 2019; Winkler et al., 2011; Winkler et al., 2013; Zhao Z. et al., 2015; Zlokovic and Apuzzo, 1997; Zlokovic, 2011). An intact and healthy B-CNS-B is essential for maintaining CNS homeostasis. However, in disease states, B-CNS-B dysfunction and disruption lead to an influx of harmful molecules from the systemic circulation into CNS, as well as cellular infiltration (Montagne et al., 2017; Sweeney et al., 2019; Zhao Z. et al., 2015; Zlokovic, 2011). These changes leave the CNS vulnerable to peripheral inflammatory derangements and may promote the progression of ALS.

A faulty barrier provides more favorable conditions for peripheral immune components to enter the CNS and makes possible an increase in communication between CNS and peripheral immunity compartments (Figure 1). Furthermore, a destruction of the barrier has been described in both ALS transgenic rodent models and ALS patients.

Garbuzova-Davis et al. (2007a) found that both the BBB and BSCB were disrupted significantly in areas of MN degeneration in an ALS transgenic mouse model (SOD1^G93A^) at multiple disease stages via electron microscope examination. This was associated with a swelling and degeneration of capillary endothelial cells, astrocytes, and MNs. However, only physical damage to the barrier structure has been shown, and it is not clear whether its proper functioning has been affected. An immunohistochemical study conducted by the same research group (Garbuzova-Davis et al., 2007b) found that there was Evans blue dye leakage in spinal cord microvessels in all of the ALS mice, indicating that endothelia and basement membranes were dysfunctional in these animals. Interestingly, three different BBB leakage markers: Evans blue, immunoglobulin G (IgG), and hemosiderin, were observed in both presymptomatic and symptomatic stages in the ALS rat model. Notwithstanding that IgG and hemosiderin could be detected in the presymptomatic stage, Evans blue extravasation, which fits best with BBB/BSCB impairment, could only be seen in the symptomatic stage (Nicaise et al., 2009). Furthermore, recently, it was reported that BSCB disruption occurred prior to MN degeneration (Winkler et al., 2014; Zhong et al., 2008). Importantly, there is some evidence confirming that the extent of MN impairment and dysfunction was proportional to the degree of BSCB breakdown in the early disease stage, as assessed in ALS mice, by using spontaneous or warfarin-accelerated microvascular lesions. Moreover, early recovery of BSCB integrity could delay the onset of MN injury and degeneration, suggesting that BSCB disruption plays a role in the onset and/or progression of ALS (Winkler et al., 2014).

Recently, it was reported that the mRNA expression of the genes *zona occludens 1* and *occludin* decreased in spinal cord tissue derived from patients with ALS, indicating that tight junction proteins could also be decreased (Henkel et al., 2009a). In addition, in line with the results of previous animal experiments, Garbuzova-Davis et al. (2012) revealed that BBB/BSCB integrity was damaged in postmortem gray and white matter of medulla and spinal cord tissue from ALS patients, as compared to healthy control patient-derived tissue. Additionally, the BSCB breakdown with erythrocyte extravasation and pericyte reduction is present in ALS patients (Winkler et al., 2013).

## Monocytes/Macrophages

In both humans and rodents, monocytes are derived from the bone marrow precursors in the bone marrow and can be divided into two main subpopulations: the “classical” CD14^++^CD16^–^ monocytes in human (Ly6C^+^ in rodents) expressing higher levels of the CCR2 and the “nonclassical” CD14^+^CD16^++^ monocytes in human (Ly6C^–^ in rodents) expressing higher levels of the CX3 chemokine receptor 1 (CX3CR1; fractalkine) (Butovsky et al., 2012; Davies et al., 2013; Hohsfield and Humpel, 2015; Mitchell et al., 2014; Yrlid et al., 2006). During inflammatory conditions, immature monocytes are recruited to the site of inflammation by CCL2 or CX3 chemokine ligand 1 (CX3CL1), where they differentiate into effector cells (including macrophages) in order to be able to exert a variety of mechanisms (Hohsfield and Humpel, 2015; Ziegler-Heitbrock, 2007).

There is mounting evidence suggesting that circulating monocytes/macrophages contribute to disease progression in transgenic rodent models of ALS. A comprehensive spatiotemporal analysis of disease progression in the ALS model SOD1^G93A^ mice demonstrated that there was denervation at the neuromuscular junction, prior to the loss of the spinal MN. This suggests that MN pathology had begun at the distal axon and proceeded in a retrograde “dying back” process (Fischer et al., 2004). Neuromuscular junctions and distal axons are located outside of the B-CNS-B and therefore can continuously access circulating monocytes/macrophages. Importantly, macrophage activation occurred presymptomatically (Chiu et al., 2009; Graber et al., 2010; Kano et al., 2012; Lincecum et al., 2010), and the monocytes/macrophages were observed to accumulate specifically and progressively along the length of the degenerating nerve fibers in muscles, sciatic nerves, and ventral roots (Chiu et al., 2009). The primary role of macrophages is thought to be the phagocytic clearance of debris, after the initial axonal degeneration event (Chiu et al., 2009). However, in ALS mice, the function of the accumulating CD 68^+^ macrophages in peripheral nerves has not been clarified (Kano et al., 2012). Moreover, it has been reported that monocytes are skewed toward a proinflammatory state, in ALS peripheral circulation, which may participate in the inflammatory response associated with disease progression (Zhao et al., 2017). Subsequently, these investigators further demonstrated that circulating monocytes derived from ALS patients were more readily activated and differentiated to an M1 phenotype, producing more proinflammatory IL-6 and TNF-α. Importantly, IL-6 protein levels and TNF-α protein levels of ALS M1 macrophages were positively correlated with the disease burden and disease progression rate, respectively (Du et al., 2020). Together, these data support the idea that peripheral monocytes/macrophages are activated in ALS and may contribute to the progression of the disease.

There is still some controversy about whether peripheral monocytes/macrophages infiltrate the CNS. A parabiosis experiment found no evidence of microglial progenitors being recruited from the circulation in either experimental denervation or CNS neurodegenerative disease (Ajami et al., 2007). In line with the above notion, another study found that the microgliosis, present in spinal cord tissue of ALS mice, results from an expansion of resident microglia (Solomon et al., 2006). However, even if the altered monocytes/macrophages cannot gain access to the CNS to regulate the course of disease directly, they could also indirectly affect central nervous inflammation by regulating the status of T cells, dendritic cells, or natural killer cells in lymph nodes, spleen, or peripheral circulation (Zhao et al., 2017).

In contrast to the above observations, there is an increasing body of evidence showing that peripheral monocytes actually migrate to CNS (Henkel et al., 2004). In rodent models of ALS, a study carried out by Butovsky et al. (2012) revealed that before the disease actually manifested itself, splenic Ly6C^hi^ monocytes expressed a polarized macrophage phenotype (M1 signature), which included increased levels of chemokine receptor CCR2. Furthermore, with the onset of the disease, microglia expressed increased levels of CCL2 and other chemotaxis-associated molecules, leading to the recruitment of monocytes into the CNS. In addition, CCL2 mRNA and immunoreactivity were up-regulated in the neurons and glial cells of ALS mice early in disease (Henkel et al., 2006). Importantly, when the researchers (Butovsky et al., 2012) treated the ALS mice with anti-Ly6C monoclonal antibody (mAb), this had the effect of reducing monocyte recruitment to the spinal cord, diminishing neuronal loss, and extending animal survival times. Accordingly, these data support the hypothesis that, in the early stages of disease, recruitment of monocytes (M1 signature) into the spinal cord has a role in MN neurotoxicity.

In ALS patients, peripheral monocytes have also been found to invade the CNS. Monocytic transcripts (CD14) were found to be increased in the spinal cord tissue of ALS patients (Henkel et al., 2004). Additionally, a recent study showed that levels of monocytes (CD14^+^) in the peripheral blood of ALS cases were significantly reduced, which may be caused by their recruitment into the CNS (Mantovani et al., 2009). In addition, emerging evidence has confirmed that there is an increased CNS infiltration by peripheral monocytes in ALS patients, as compared with healthy age-matched controls (Zondler et al., 2016). Furthermore, it has been reported that application of human immunoglobulins or fusion proteins increased CNS invasion of peripheral monocytes and delayed disease onset, indicating that peripheral monocytes have a protective role in the early phase of ALS (Zondler et al., 2016).

As indicated above, peripheral monocytes/macrophages are activated in ALS and can infiltrate into the CNS, affecting the progression of the disease. However, whether their effects on the ALS progression are beneficial or harmful is still debatable; therefore, future research needs to provide stronger evidence for these effects.

## T Cells

T cells, derived from lymphoid stem cells in bone marrow, differentiate and mature in thymus and reach the peripheral immune organs through the bloodstream, where they play a variety of roles. CD4^+^ T cells and CD8^+^ T cells can be distinguished according to the CD molecules that they express.

## CD4^+^ T Cells

CD4^+^ T cells are mainly helper T cell (T_H_) subsets, which generally exert their auxiliary and effector functions through the synthesis and secretion of cytokines. According to the different cytokines produced, they can be divided into T_H_1 cells, T_H_2 cells, T_H_17 cells, and Tregs, among other subtypes. T_H_1 cells and T_H_17 cells promote neuroinflammation, because they are producers of proinflammatory cytokines and enhance microglia-mediated neurotoxic effects, by up-regulating the release of ROS and NO. Conversely, T_H_2 cells and Tregs, as producers of anti-inflammatory cytokines, enhance microglia-mediated neuroprotective effects (Gendelman and Appel, 2011).

The circulating CD4^+^ T cells are involved in ALS pathology via a variety of mechanisms. It has been demonstrated that the number of Tregs in the blood of ALS patients negatively correlates with the rate of disease progression (Beers et al., 2011a; Rentzos et al., 2012). Noteworthy is the fact that Tregs acquire an immunosuppressive ability via their high-level expression of the transcription factor (TF), Foxp3, a Treg-specific TF, suggesting that this TF is a key molecule in mediating a suppression type phenotype (Fontenot et al., 2003; Hori et al., 2003; Khattri et al., 2003; Sakaguchi et al., 2008; Sakaguchi et al., 2009). Specifically, levels of Foxp3 in the blood were predictive of the progression rate and survival (Henkel et al., 2013). In addition, compared with the control groups, Tregs derived from peripheral blood of ALS patients have a poor ability in inhibiting the proliferation of T lymphocytes; that is, the greater the disease burden or the faster the disease progression as assessed by clinical evaluation, the greater the level of Treg dysfunction (Beers et al., 2017).

In addition to affecting ALS progression via the circulating CD4^+^ T cells, peripheral CD4^+^ T cells can infiltrate into the CNS and affect ALS progression. In two-thirds of ALS patients, perivascular and intraparenchymal CD4^+^ T cells have been detected near the degenerated corticospinal tract and ventral horn, demonstrating that CD4^+^ T cells can infiltrate into CNS during the course of the disease (Engelhardt et al., 1993). Besides, several studies have demonstrated that the CNS infiltration of CD4^+^ T cells may be associated with increased levels of CCL2 and the activation of microglia at the morphological level (Beers et al., 2017; Henkel et al., 2004; 2006; 2009b). Furthermore, significantly increased expression of CXCR3, CXCR4, CCL2, and CCL5 was evident on T cells taken from ALS patients, as compared to healthy controls (Perner et al., 2018). These reports indicate that the recruitment and infiltration of CD4^+^ T cells into the CNS are a critical event in ALS.

Recent work has highlighted the key role of these infiltrating CD4^+^ T cells in protecting MNs, in ALS patient spinal cords. As the protective effect of CD4^+^ T cells on MNs is more widely recognized and less controversial than that of the other immune cells, there is a sense of urgency that studies should be initiated to address the potential and understand the exact mechanisms by which CD4^+^ T cells are able to slow down the progression of this disease.

In 2008, several research groups detailed the protective effects of CD4^+^ T cells in rodent ALS models. Chiu et al. (2008) found that CD4^+^ T cells infiltrated into the spinal cord of SOD1^G93A^ mice during disease progression, and the disease was significantly accelerated when mutant SOD1 mice were propagated in a TCRβ deficient background. Beers et al. (2008) found that, when mSOD1 mice were bred with mice lacking functional T cells or CD4^+^ T cells, the progression of MN disease was accelerated; however, reconstituting mice with T cells via bone marrow transplants resulted in prolonged survival. Banerjee et al. (2008) found that the passive transfer of *ex vivo* activated CD4^+^ T lymphocytes into mSOD1 mice, delayed neurological symptom onset, and extended the animals’ survival. When mSOD1 mice were bred with T-cell–deficient mice, the levels of neurotrophic factor, glial glutamate transporter, and IGF-1 in the spinal cord were decreased, but at the same time, the levels of proinflammatory cytokines and NOX2 were increased (Beers et al., 2008; Chiu et al., 2008). These findings show that reconstruction of CD4^+^ T-cell populations in animals can confer a degree of neuroprotection by regulating the glial balance between neurotrophic effect and cytotoxicity and that the immune response mediated by CD4^+^ T cells/microglia interactions plays a vital neuroprotective role in ALS mice (Beers et al., 2008; Chiu et al., 2008).

After the protective effects of CD4^+^ T cells were clarified, further studies were conducted on Tregs, one of the CD4^+^ subgroups. In the absence of *ex vivo* activation, endogenous Tregs from ALS mice, in the early stage of disease, were passively transferred into ALS mice lacking functional T lymphocytes, which prolonged the recipients’ disease duration and survival, highlighting the protective effect of endogenous Tregs. Furthermore, they revealed that the neuroprotective effect of Treg was mediated by the increased secretion of IL-4, which can directly inhibit the toxicity of microglia (Beers et al., 2011a; Figure 1). Moreover, the mechanisms of Tregs inhibiting the activation of microglia in ALS model have also been explored through *in vitro* experiments. When cocultured with mSOD1 adult microglia, isolated mSOD1 Tregs, from the spleen and lymph nodes, inhibited microglial cytotoxic cytokines, as well as NOX2, and induced nitric oxide synthase expression by secreting IL-4 (Zhao et al., 2012; Figure 1). It is worth noting that, in the previous paragraphs, we have stated that inhibition of microglia toxicity can lead to a prolongation of survival in mouse models of ALS. A recent study found that the amplification of effector Tregs in SOD1^G93A^ mice, through IL-2c infusions, was associated with significantly slower disease progression and longer survival. Interleukin 2c treatment resulted in marked invasion of T cells into the ventral horns of spinal cords. This study is unique in that the intervention was an *in vivo* amplification of the host’s Tregs, rather than the *in vitro* transplantation of Tregs. More importantly, the *in vivo* amplification of Tregs was associated with the retention of somatic cell size of spinal MNs in SOD1^G93A^ mice, as well as a significant inhibition of immunoreactivity of astrocytes and microglia and also increased expression of neurotrophic factor genes in both spinal cord and peripheral nerves (Sheean et al., 2018).

In addition to interacting with glial cells, Tregs are also able to communicate with Teffs to influence the progression of the disease. In the stable stage of the disease, the total number of Tregs in ALS mice increased. It was found that Tregs isolated at this stage (from the spleen and lymph nodes) inhibited Teffs proliferation by secreting IL-4, IL-10, and TGF-β (Figure 1). On the contrary, in the rapid stage of the disease, the number of mSOD1 Tregs decreased, whereas the proliferation of mSOD1 Teffs increased (Zhao et al., 2012).

As the disease progresses, it has been reported that there is a shift from a supportive Tregs/M2 response to an injurious T_H_1/M1 response (Zhao et al., 2013). Regulator T cells have been shown to directly induce the differentiation of macrophages and microglia into an M2-activated state (Liu et al., 2011; Tiemessen et al., 2007; Figure 1). In turn, M2 microglia induced the production of Tregs that were capable of inhibiting Teffs (Savage et al., 2008; Figure 1). T_H_1 cells are known to produce interferon γ (IFN-γ), which promotes the activation of M1 type microglia (Figure 1). In turn, M1 microglia can promote the proliferation and function of T_H_1 cells (Gao and Tsirka, 2011; Zhao et al., 2013; Figure 1). In the early, slowly progressing, stages of the disease, Tregs accumulate in the blood and lymph nodes, whereas the expression of Foxp3 mRNA in spinal cord is induced. When the disease rapidly accelerated, the numbers of Treg declined, which could possibly be mediated through the loss of Foxp3 expression in these cells (Beers et al., 2011a). In addition, both T_H_1 cells and T_H_17 cells release large amounts of TNF-α, a compound known to induce Treg dysfunction by suppressing the phosphorylation of Foxp3 (Nie et al., 2013; Figure 1). Additionally, a study conducted with the ALS mouse model showed that the neurotoxic effects were associated with elevated levels of IL-6 released by T_H_1 cells (Beers et al., 2011b). Interleukin 6 has been reported to contribute to inhibiting the production of Foxp3^+^ Tregs (Bettelli et al., 2006; Figure 1).

## CD8^+^ T Cells

CD8^+^ T cells, also known as cytotoxic T lymphocytes, can exert their effects by releasing cytotoxic proteins or by triggering programmed cell death. They can also participate in the regulation of the immune response by releasing cytokines such as IFN-γ and TNF-α.

CD4^+^ T cells have been observed in the spinal cord during all phases of the disease. However, CD8^+^ T cells were present only at the end stage of the disease and made up only a small fraction of the total T-cell population (Beers et al., 2008; Chiu et al., 2008; Henkel et al., 2006; Zhao et al., 2013). Because of their small numbers, the role of CD8^+^ T cells has been largely ignored by previous investigators. However, recently, CD8^+^ T cells have gradually started to attract more attention.

Recently, it was suggested that circulating CD8^+^ T cells play a key role in ALS. Damaged MNs in slow-progressing ALS mice actively recruit CCL2, MHC-I, and complement C3, and this was accompanied by an intense infiltration of CD8^+^ T cells and macrophages in the peripheral nervous system (PNS). Following infiltration, CD8^+^ T cells and macrophages are capable of promoting the regeneration of myelin along the motor axon and at the neuromuscular junction, thus delaying muscle denervation and prolonging survival. On the contrary, a lack of this response was shown to negatively affect the course of the disease (Nardo et al., 2016). Clearly, it is now evident that CD8^+^ T cells should not be stereotyped as only being neurotoxic; instead, they can protect MNs in specific situations.

Emerging evidence has confirmed that CD8^+^ T cells can infiltrate into the CNS of both ALS patients and SOD1 mice (Chiu et al., 2008; Chiu et al., 2013; Holmoy, 2008; Sta et al., 2011). The infiltrated CD8^+^ T cells have been classically considered as being detrimental for MNs. For example, MHC-I depletion in resident microglia and the lack of CD8^+^ T cell infiltration in the spinal cord protected cervical MNs, thus delaying the paralysis of forelimbs and prolonging the life span of SOD1^G93A^ mice (Nardo et al., 2018).

As mentioned previously, it has been claimed that the presence of CD8^+^ T cells in spinal cord is neurotoxic to MNs. However, the exact mechanism is not yet clear. A study carried out by Coque et al. (2019) demonstrated a specific MHC-I–dependent interaction between MNs and SOD1^G93A^ CD8^+^ T cells; these investigators measured the interaction strength using atomic force microscopy-based force spectroscopy at the single-cell level. They also demonstrated that activated mutant SOD1 CD8^+^ T cells produced IFN-γ, which elicited the expression of the MHC-I complex in MNs, allowing themselves to exert a cytotoxic function through the Fas and granzyme pathways.

## Immune-Related Component: Gut Microbiota

With the emerging popularity of the hypothesis that there is immune dysregulation involved in ALS, there is a need to step forward from just talking about changes in the traditional immune components and instead take the emerging immune-related components into account. In the field of neurology, the role of the gut microbiota has become a hot research topic in recent years. Although the CNS appears to be separated from the rest of the body by the presence of the B-CNS-B, many studies have revealed interactions between the CNS and gut microbiota (Erny et al., 2015). Gut microbiota serves as an intersection between the microbiome–gut–brain axis and the neuroimmune-endocrine axis (Zhong et al., 2019). Additionally, 70–80% of the total number of human immune cells are the immune cells within intestinal mucosal lymph nodes (Fanous et al., 2007). These results suggest that gut microbiota may possess the ability to exert an impact upon neuroimmunity.

It is not fully understood how gut microbiota and its components (ranging from whole or parts the microorganisms themselves, to the metabolites they produce, either themselves or via their actions on diet-procured molecules) are able to control CNS immune response in distant sites. We propose two possible pathways. One is that gut microbiota or its components are able to infiltrate into the peripheral circulation and influence the amount, migration, and function of peripheral immune components and, in that way, affect the crosstalk between the periphery and the CNS. The second alternative is that gut microbiota or its components themselves can cross the damaged B-CNS-B and directly undergo a crosstalk with immune components in the CNS. The following data might provide some basis to support both these hypotheses. Under pathological conditions, evidence from several studies has emerged that gut microbiota or its components translocated into the bloodstream or CNS. *Escherichia coli* were inoculated into SPF mice via oral gavage after a surgically induced stroke; subsequently, the numbers of these bacteria were found to be significantly elevated in peripheral blood. These data demonstrated that gut microbiota can translocate into bloodstream when the intestinal vascular permeability was evident (Stanley et al., 2016). Furthermore, the presence of LPS in the blood circulation was detected in stressed rats (Ait-Belgnaoui et al., 2012). Recently, Singer et al. (2018) found that viable gut-associated bacteria were present in the brains of sepsis-survivor mice, suggesting that sepsis had allowed the bacteria to spread into the brain. Moreover, it was reported that dietary tryptophan was metabolized by the gut microbiota into indoxyl-3-sulfate and that this compound crossed the BBB and acted on astrocytes (Rothhammer et al., 2016). The administration of *Roseburia intestinalis* or a rationally selected mixture of *Clostridia* strains was able to increase the proportion of Tregs and attenuate colitis (Atarashi et al., 2013; Zhu et al., 2018). Furusawa et al. (2013) revealed the mechanisms by which *Clostridia*-derived butyrate is able to induce the differentiation of Tregs. Furthermore, recent study showed that gut microbiota–derived bacterial fermentation products, short-chain fatty acids (SCFAs), can regulate Treg homeostasis in an Ffar2-dependent manner (Smith et al., 2013). *Citrobacter rodentium* was reported to be involved in regulating the number of T_H_17-positive cells (Ryz et al., 2012). Gut microbiota can stimulate chemokine expression, which favors enhanced recruitment of T cells (Cremonesi et al., 2018). It was claimed that the elevated level of endotoxin/LPS, that is, a compound synthesized by microorganisms, in the plasma of patients with ALS was positively correlated with blood monocyte/macrophage activation (Zhang et al., 2009). In addition, tolerable low doses of LPS have been shown capable of activating a neuroprotective effect from microglia (Chen et al., 2012). Host microbiota target microglia to regulate their maturation and function and shape CNS immunity via the SCFAs (Erny et al., 2015). Gut microbiota metabolize dietary tryptophan into aryl hydrocarbon receptor agonists that suppress neuroinflammation by activating aryl hydrocarbon receptor–dependent signaling in astrocytes (Rothhammer et al., 2016).

There is accumulating evidence suggesting that changes in the gut microbiota possess a strong connection with neurodegenerative diseases, in particular Parkinson disease (Lin et al., 2018; Sampson et al., 2016; Scheperjans et al., 2015), Alzheimer disease (Harach et al., 2017; Paley et al., 2018; Zhuang et al., 2018), and multiple sclerosis (Jangi et al., 2016). Amyotrophic lateral sclerosis is also a neurodegenerative disease, and neuroinflammation is one of its common characteristics with the aforementioned diseases. Even though studies on the relationship between gut microbiota and ALS are still at an early phase, the phenomenon in which the gut microbiota has been closely associated with other neurodegenerative diseases raises hopes about the future.

We hypothesized that gut microbiota could affect the progression of ALS. To support that notion, one study found that there was a damaged tight junction structure and increased intestinal permeability of SOD1^G93A^ mice model and an increased number of abnormal intestinal Paneth cells, which play key roles in host innate immune responses. The aforementioned changes were associated with decreased levels of butyrate-producing bacteria (i.e., *Butyrivibrio fibrisolvens*, *E. coli*, and *Fermicus*) in SOD1^G93A^ mouse feces (Wu et al., 2015). After SOD1^G93A^ mice were administered with 2% butyrate, intestinal microbial homeostasis was restored, gut integrity was improved, the numbers of abnormal Paneth cells were significantly decreased, and life span was prolonged. Butyrate treatment has been shown to be successful in decreasing aggregation of the SOD1^G93A^ mutated protein in both ALS mice and intestinal epithelial cells cultured from humans, indicating that this compound can restore intestinal homeostasis and microbiota (Zhang et al., 2017). In SOD1^G93A^ mice, alterations in the gut microbiota preceded the onset of motor symptoms and muscle atrophy. With these changes in the gut microbiota and neuromuscular deterioration, the immune cells also expanded and were activated. More importantly, a correlation analysis revealed that the size of tibialis anterior muscle in ALS mice correlated positively with the numbers of Lachnospiraceae. The correlation analysis also revealed potential associations between the gut microbiota and neuroinflammation. These data suggested that gut microbiota can influence neuroinflammation and thus ALS progression (Figueroa-Romero et al., 2019). Just a few months ago, an article in the journal *Nature* reported that, by exploiting transgenic mice models, researchers had identified several intestinal bacteria associated with the severity of ALS: It was found that while *Ruminococcus torques* and *Parabacteroides distasonis* exacerbated the symptoms of ALS, *Akkermansia muciniphila* ameliorated them. Consequently, when they administered *A. muciniphila* to ALS mice, it was observed that the *A. muciniphila*–associated nicotinamide accumulated in the CNS. Furthermore, they demonstrated that systemic supplementation of nicotinamide improved motor symptoms and gene expression patterns in the spinal cord of ALS mice; this may usher in new therapeutic strategies for ALS treatment (Blacher et al., 2019). In another study, Fang et al. (2016) found that host microbiota were markedly different in health and disease. For instance, the genus *Dorea* was overexpressed in ALS patients, whereas *Anaerostipes*, *Lachnospira*, and *Oscillibacter* were less extensively expressed as compared to controls. A comprehensive examination of fecal microbiota in five patients with ALS and MN disorders revealed that all five patients displayed alterations in their gut microbiota, characterized by a lower species diversity, as compared to healthy controls with relatively intact abundance (Rowin et al., 2017). Another study with a larger sample size, examining 50 ALS patients and 50 healthy controls, found that the abundance of *E. coli* and enterobacteria was higher, but the abundance of total yeasts was lower in the patients as compared to samples derived from the controls. However, the authors speculated that there was no evidence of substantial dysbiosis status in ALS patients, even though there were differences between healthy people and patients (Mazzini et al., 2018). In view of these promising results, a randomized, multicenter, double-blind clinical trial (NCT03766321) employing fecal microbial transplantation (FMT) as a therapeutic intervention for ALS patients was recently initiated. In this clinical trial, the researchers will primarily monitor changes in the Treg number between FMT-treated and control subjects. They expect FMT to increase the number of Tregs and re-establish the neuroprotective microenvironment surrounding MNs. The results have not been published (Mandrioli et al., 2019).

Although the above results point to a possible correlation between gut microbiota and the progression of ALS, there are several studies that show conflicting results. One study was done by comparing the fecal microbiota of 25 ALS patients with 32 age- and gender-matched healthy persons and the abundance and the diversity of the bacterial taxa at different taxonomic levels, and PICRUSt-predicted metagenomes were almost indistinguishable, suggesting that ALS patients do not exhibit a real alteration in the composition of their gut microbiota (Brenner et al., 2018). The inconsistencies in these results may be explained in part by the fact that the latter study enrolled more subjects with well-defined clinical characteristics and applied a more stringent statistical analysis. Nonetheless, the possibility should not be excluded that the microbiota varies, depending on geography and diet (Brenner et al., 2018).

As described above, whereas some studies have suggested a link between gut microbiota and ALS, others have come to the opposite conclusion. The inconsistency in results may be due to differences in sample sources, experimental techniques, and statistical methods. Clearly, there is an urgent need to explore the role of gut microbiota in ALS and the relative mechanisms, in order to clarify the situation. More research needs to be conducted in order to identify if there is any specific genus(es) present in the microbiota that play a central role in modulating this immune crosstalk. Therefore, it is possible that application of specific microorganisms may lead to new therapies for ALS in the future.

## Discussion

This review summarizes our current knowledge of the mechanisms that contribute to neuroinflammation in ALS. Previously, most studies have focused on the relationship between CNS immune response and MN degeneration. Until now, there has been little information available regarding the ability of the peripheral immune response to modify the survival of MN. So far, it seems that no single immune component is able to control the fate of ALS or to reverse its course. Increasing evidence points to a complex crosstalk between the CNS and peripheral immune components. As our understanding of this conversation deepens, novel ideas will arise following basic and applied science to test their applicability toward ALS treatment.

Whether microglia exert neuroprotective or neurotoxic function depends on the different states of microglial activation influenced by local factors and stimuli. Hence, future explorative studies could focus on the modulation of these factors and stimuli, in order to promote M2 polarization of microglia in a targeted manner.

As with microglia, peripheral monocytes/macrophages also possess both neuroprotective and neurotoxic potentials, and enhancing their anti-inflammatory response and inhibiting their proinflammatory response may be an important future direction. Additionally, the role of monocytes/macrophages in the peripheral immune response should not be underestimated. Whether they act on the distal axon and neuromuscular junction or affect the immune status of peripheral circulation may change the prognosis of ALS patients.

As things stand, Tregs have the best recognized neuroprotective function and can protect MNs in both CNS and PNS. In this context, they deserve the most attention. Regulator T cells exert their neuroprotective function through various mechanisms as mentioned previously. They can significantly regulate effector cells, such as microglia, Teffs, and MNs, so as to maintain the anti-inflammatory state of the microenvironment around MNs and facilitate their survival. Regulator T cells have been recognized as one of the most promising candidates for immune-cell therapy, in the light of positive results from multiple animal trials and clinical trials (Sheean et al., 2018). However, more basic studies, exploring the exact mechanisms and multicenter randomized controlled trials enrolling more subjects, will be needed to confirm this concept.

Recently, interest in CD8^+^ T cells has increased, as they are no longer considered as being merely toxic toward MNs. These cells have been shown to promote the myelin regeneration of motor axons and neuromuscular junctions in the peripheral nervous system to prolong survival. In addition, considering the different roles of CD8^+^ T cells in the central and peripheral, it is possible that the disease should be treated at different times at different locations. Inhibition of central CD8^+^ T cells and activation of peripheral CD8^+^ T cells might represent a potential route for improving patient outcomes. Nevertheless, as the number of infiltrated CD8^+^ cells in CNS is small, there has been little information available regarding the role of CD8^+^ T cells in the progression of ALS; therefore, more advanced detection techniques and more accurate experimental protocols need urgently to be developed.

Increasing knowledge about gut microbiota–brain bidirectional communication has provided new insights into understanding many complex neurological disorders. As the knowledge in this field grows, researchers have found that the gut microbiota is involved in a variety of neurological diseases. Nevertheless, there is still not enough evidence to state that a relationship exists between the gut microbiota and ALS. Therefore, considerably more work needs to be done in this area to determine whether the gut microbiota of ALS patients has changed. If it can be proven that these changes have occurred, the next challenge would be to identify the key bacteria most likely to influence the immune dysregulation present in ALS. The ultimate goal would be to change the PNS and even CNS immune status by regulating this key bacterial component or its metabolic products, as a novel way of prolonging the survival of ALS patients.

Although great strides have been made toward improving the clinical symptoms and prognosis of model animals, there is still a long way to go before successful clinical translation can be achieved. To date, no neuroinflammation-related therapy has been approved for the treatment of ALS patients because relevant clinical trials did not turn out as expected. Why are the positive results from animal tests not capable of being replicated in patients? What could be soon translated into clinical research that might ultimately benefit patients?

First, the transgenic mouse model cannot completely replicate the pathological changes of ALS patients. In the future, if there are cell and animal models that bear greater similarities to the pathology of patients, then research on ALS will surely advance.

Second, the evidence presented in this review indicates that the course of ALS can be roughly divided into two stages: microglia and T cells play a neuroprotective role in the early stage of the slow progression, but they both convert to display neurotoxic phenotypes after the disease has entered into the advanced stage with a rapid progression. Hence, defining the stage-dependent treatment strategy, that is, the appropriate treatment according to the different pathology at different stages, may provide a pathway toward improved treatment of ALS.

Last but not least, our work emphasizes that the pathogenesis of ALS is a complex interlaced network. The therapeutic impact of targeting a single factor is likely to be minimal and will most likely to be offset by intercellular interactions. Instead, cell therapy targeting multiple factors and communicating with multiple host cell types will hopefully overcome the difficulties. Because eliminating or killing proinflammatory cells in the body can have many unpredictable and detrimental effects, increasing anti-inflammatory cells through passive transfer or *in vivo* amplification seems to be a more viable strategy. Regulator T cells have the clearest application prospects in terms of their neuroprotective effects at all stages of the disease, and the preclinical data supporting this role are both abundant and comprehensive. Additionally, other beneficial cell subtypes, such as M2 microglia, presymptomatic astrocytes, and stem cells, are also worth examining in greater detail.

In conclusion, it has become obvious that the problem of neuroinflammation lies not only in CNS. Instead of focusing exclusively on either peripheral immunity or CNS immunity, but rather on the dialogue between them is the major innovation we present in this review. Peripheral monocytes/macrophages, T cells, gut microbiota, or its products can infiltrate into the CNS and directly interact with MNs or the surrounding glial cells, potentially either protecting or damaging MNs.

The road to a feasible and effective therapy for ALS has been long and overshadowed by major setbacks. In this extremely complex disease, the use of therapies to treat patients has been largely unsuccessful because of our failure to grasp how the immune crosstalk affects disease initiation and progression. Therefore, a detailed understanding of each key step of the infiltration process from the peripheral circulation to the CNS and the positive or negative intervention of these steps may provide new insights toward the treatment of ALS. Consequently, targeting the crosstalk between the CNS immune response and peripheral immune response is likely to become one of the next frontiers in the treatment of ALS.

## Author Contributions

ZL had the idea for this review, performed the literature search and drafted the manuscript. XC and SZ revised the manuscript. XZ, CL, FL, and CZ proofread the manuscript. All authors contributed to the manuscript and approved the submitted version.

## Conflict of Interest

The authors declare that the research was conducted in the absence of any commercial or financial relationships that could be construed as a potential conflict of interest.

## Abbreviations

ALS: amyotrophic lateral sclerosis
B-CNS -B: blood-central nervous system barrier
BBB: blood–brain barrier
BSCB: blood–spinal cord barrier
CNS: central nervous system
CTLs: cytotoxic T lymphocytes
IFN-: interferon-
IGF-1: insulinlike growth factor-1
IL-: interleukin-
iNOS: induced nitric oxide synthase
MHC-: major histocompatibility complex-
MN: motor neuron
NF- κ B: nuclear factor-kappa B
NO: nitric oxide
PET: positron emission tomography
ROS: reactive oxygen species
SCFAs: short chain fatty acids
TF: transcription factor
T_H_: helper T cell
TNF: TUMOR necrosis factor
Tregs: regulator T cells.
